# Hemodynamic changes in supra-aortic trunks after transcatheter aortic valve implantation at duplex ultrasound examination

**DOI:** 10.1093/ehjimp/qyaf151

**Published:** 2025-12-03

**Authors:** Rocco Pasqua, Giampaolo Luzi, Gianluca Paternoster, Danilo Menna, Elena Orlando, Vincenzo Fioretti, Priscilla Nardi, Giulio Illuminati, Vito D’Andrea, Eugenio Stabile, Andrea Esposito

**Affiliations:** Cardiovascular Department, San Carlo Hospital, Via Matera 4, Potenza 85100, Italy; Department of Surgery, Sapienza University of Rome, Rome, Italy; Cardiothoracic and Vascular Department, San Camillo Forlanini Hospital, Rome, Italy; Cardiovascular Department, San Carlo Hospital, Via Matera 4, Potenza 85100, Italy; Department of Health Science Anesthesia, University of Basilicata, Potenza, Italy; Cardiovascular Department, San Carlo Hospital, Via Matera 4, Potenza 85100, Italy; Radiology Department, San Carlo Hospital, Potenza, Italy; Cardiovascular Department, San Carlo Hospital, Via Matera 4, Potenza 85100, Italy; Department of Surgery, Sapienza University of Rome, Rome, Italy; Department of Surgery, Sapienza University of Rome, Rome, Italy; Department of Surgery, Sapienza University of Rome, Rome, Italy; Cardiovascular Department, San Carlo Hospital, Via Matera 4, Potenza 85100, Italy; Department of Health Science Anesthesia, University of Basilicata, Potenza, Italy; Cardiovascular Department, San Carlo Hospital, Via Matera 4, Potenza 85100, Italy

**Keywords:** aortic stenosis, internal carotid artery stenosis, aortic valve repair, surgical aortic valve replacement, TAVI, duplex ultrasound

## Abstract

**Aims:**

The hemodynamic consequences of aortic stenosis (AS) on supra-aortic trunks may play a potential role during the diagnosis of concomitant internal carotid artery (ICA) stenosis by dampening blood flow velocity. To investigate the effect of AS on ICA blood flow we evaluated carotid and vertebral blood flow velocity indexes in patients undergoing transcatheter aortic valve implantation (TAVI).

**Methods and results:**

Patients admitted for endovascular treatment of a severe AS underwent supra-aortic duplex ultrasound examination prior and after TAVI to be enrolled in the study. Patients with symptomatic or severe ICA stenosis were excluded. Patients with other cardiac impairments that could configure a confounding factor were excluded. One hundred and five patients of a median age of 80 years met the study inclusion criteria. The median peak systolic velocity (PSV) of the assessed supra-aortic arteries increased after TAVI: common carotid artery (CCA) from 64.5 to 78.0 cm/s (+24%; *P* < 0.01), ICA from 67.0 to 90.5 cm/s (+36%; *P* < 0.01), and vertebral artery (VA) from 44.0 to 51.0 cm/s (+17%; *P* < 0.01). Median end-diastolic velocity (EDV) also increased: CCA from 12.0 to 14.0 cm/s (+12%; *P* < 0.01), ICA from 19.0 to 23.0 cm/s (+20%; *P* < 0.01), and VA from 10.0 to 11.0 cm/s (+18%; *P* < 0.01). In parallel, median acceleration time (AT) decreased markedly at each site: CCA from 0.180 to 0.100 s (−44%; *P* < 0.01), ICA from 0.195 to 0.100 s (−41%; *P* < 0.01), and VA from 0.180 to 0.100 s (−36%; *P* < 0.01).

**Conclusion:**

Severe AS significantly affects supra-aortic arteries blood flow as assessed by duplex, by decreasing both PSV and EDV and increasing AT. This study suggests that carotid ultrasound criteria to assess ICA stenosis severity should be re-evaluated in larger multi-centre studies to validate their predictive values in patients with concomitant AS.

## Graphical Abstract

**Graphical Abstract:**
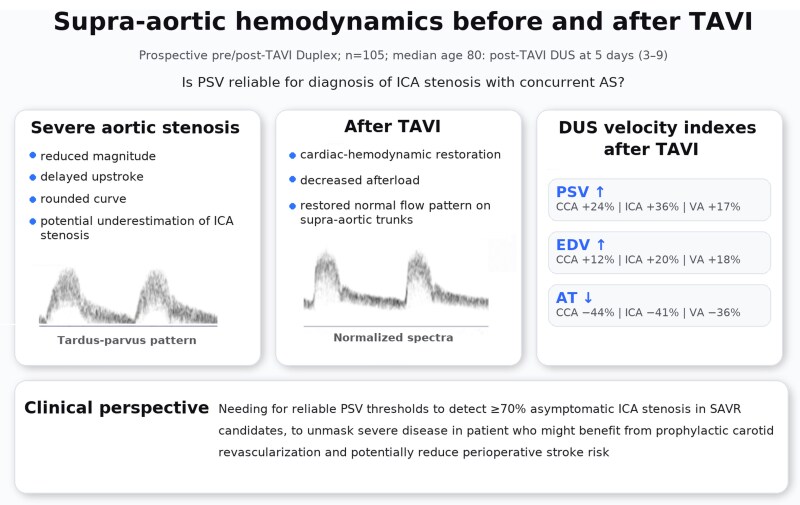
TAVI, transcatheter aortic valve implantation; DUS, duplex ultrasound; PSV, peak systolic velocity; ICA, internal carotid artery; AS, aortic stenosis; EDV, end-diastolic velocity; AT, acceleration time; SAVR, surgical aortic valve replacement

## Introduction

Aortic stenosis (AS) is the most frequent heart valve disease in the elderly population and its prevalence is rising as a consequence of the ageing of the population. Prevalence of a concomitant internal carotid artery (ICA) stenosis ≥ 50% ranges from 13% to 33%^1,2^ and coexistence of both conditions may have a role over neurological outcomes of patients undergoing open surgical treatment for AS. Duplex ultrasound (DUS) is the gold standard for diagnosis of ICA stenosis by visualization of the plaque and blood flow velocity assessment, the latter being the mainstay for grading obstruction severity. A peak systolic velocity (PSV) > 230 cm/s is considered the threshold for severity.^3^ The increased afterload of the left ventricle determined by the presence of AS results in an abnormal flow pattern distal to the aortic valve, which is characterized by a delayed upstroke, a rounded curve of the waveform with reduced magnitude. The typical blood flow pattern of the AS is evident at duplex examination of the supra-aortic arteries^4,5^ and may contribute to underestimation of carotid artery stenosis.^6^ Since transcatheter aortic valve implantation (TAVI) theoretically restores cardiac hemodynamic, can be considered a clinical model to investigate the role of AS on supra-aortic arteries blood flow duplex mediated assessment. The aim of this prospective single-arm before–after study was to evaluate the effect of severe AS on carotid and vertebral hemodynamic indexes at DUS before and after TAVI.

## Materials and methods

### Study population

This study was approved by the institutional ethics committee (CEUR-CET/CEL 20240018953) and informed consent was obtained from all participants. From January 2022 to December 2024, 212 consecutive adult patients with severe AS, high surgical risk or aged ≥75 years and suitable for TAVI at the Division of Cardiology of the Azienda Ospedaliera San Carlo, Potenza, Italy, underwent supra-aortic DUS examination prior to TAVI as a part of the pre-operative assessment and after the procedure for the purpose of the study. Severe AS is defined by the presence of an aortic valve area (AVA) < 1 cm^2^ and/or mean transvalvular aortic gradient >40 mmHg and/or peak aortic jet velocity ≥ 4 m/s according to current guidelines.^7^ Patients with reduced left ventricular ejection fraction (LVEF <50%), moderate or severe aortic valve regurgitation before TAVI, more than mild paravalvular leak after TAVI, uncontrolled blood pressure, tachyarrhythmias occurring before and/or after TAVI, severe anaemia or hyperthyroidism were excluded from the registry to avoid other potential confounding factors that may alter carotid or vertebral blood flow. Patients with symptomatic or severe ICA stenosis or carotid occlusion were excluded from this study. Inclusion and exclusion criteria are represented in the flowchart in see Supplementary data online, appendix.

### Cardiac ultrasound

All echocardiographic studies, before and after TAVI, were performed on a Vivid E95 (GE Healthcare, Chicago, IL, USA) by a single certified physician. AVA was calculated by the continuity equation, mean gradient by the simplified Bernoulli equation, and left ventricular ejection fraction by the biplane Simpson method according to current guidelines.^8^ Doppler velocity index was measured by dividing the time-integral velocity in the left ventricular outflow tract by the time-integral velocity in the aorta. The severity of regurgitation of native valve before TAVI and paravalvular leak after the procedure was assessed by a multi-parametric approach and according to current guidelines.^9,10^ All post-operative echocardiograms were performed the day after the TAVI procedure.

### Duplex assessment

A single certified physician conducted all DUS examinations using a General Electric LOGIQ E10 ultrasound system (GE Healthcare, Chicago, Illinois, USA) with a clear linear array probe. All carotid examinations were performed with grayscale, colour Doppler, and spectral Doppler velocity determination with an insonation angle between 40° and 60°. PSV, end-diastolic velocity (EDV) and acceleration time (AT) were measured in the common, internal and vertebral artery (VA) of both sides prior and after TAVI. Values were averaged over five cardiac cycles as provided by the ultrasound system. Data were collected separately for the right and left side. ICA stenoses were classified according to duplex ultrasonographic criteria as follows. A stenosis of <50% was diagnosed in the presence of a PSV of the ICA below 125 cm/s associated with visible plaque or intimal thickening. A moderate stenosis (50–69%) was defined by the presence of plaque at grayscale and colour Doppler ultrasonography combined with an ICA PSV between 125 and 230 cm/s. Severe stenosis (≥70% but less than near-occlusion) was diagnosed when grayscale and colour Doppler ultrasonography demonstrated plaque and luminal narrowing, and the ICA PSV exceeded 230 cm/s, in accordance with the criteria of the Society of Radiologists in Ultrasound Consensus Conference. The diagnosis of near-occlusion or complete ICA occlusion was primarily based on the demonstration of a markedly narrowed lumen at colour or power Doppler imaging and was subsequently confirmed by computed tomographic angiography.^4,11,12^

### TAVI Procedure

All patients underwent TAVI by experienced physicians. The choice of valve type (self-expanding or balloon-expandable), size and access site were at the discretion of the Heart Team considering the comorbidities and the results of pre-procedural computed tomography. Only patients who underwent a successful TAVI defined by a post-operative echocardiographic transaortic mean gradient < 20 mmHg, peak velocity < 3 m/s, Doppler velocity index > 0.25, and less than moderate paravalvular leak were enrolled in our study.^13^

### Statistical analysis

Continuous variables are expressed as median and inter-quartile range (IQR). Categorical variables are expressed as numbers and percentages. Normal distribution of variables was assessed using the Shapiro–Wilk test. Within-subject pre–post comparisons used the Wilcoxon signed-rank test. We reported the within-subject change, Δ=post−pre, as median [IQR]; the percent change (Δ%) = 100 × (post/pre-1) as median [IQR]. *P*-values < 0.05 were considered statistically significant. Missing data were handled by complete-case analysis of paired observations (overall missingness <5%). The sample-size calculation targeted the within-subject percent change in ICA PSV as the primary endpoint. We tested the one-sided hypothesis that the mean percent increase would exceed 15%. The 15% threshold was based on the systematic review by Jahromi *et al.*, in which PSV values <200 cm/s were associated with a very low probability of ≥70% carotid stenosis.^14^ Accordingly, an increase from 200 to 230 cm/s (≈15%) was deemed clinically meaningful. Assuming an SD of %Δ of 39.2%, a paired one-sided *t*-test at α=0.05 with 95% power requires *n* = 75 pairs to detect a +15% mean increase. With *n* = 105 paired patients, the minimal detectable mean increase is 8.9%, and power to detect a 15% increase exceeds 99%. For the side-specific sub-analysis, right and left side measurements changes were tested with Wilcoxon signed-rank, and the between-side comparison used Wilcoxon on the paired differences. Jamovi software (The Jamovi project 2024, version 2.5) was utilized for calculations.

## Results

**Figure 1:**
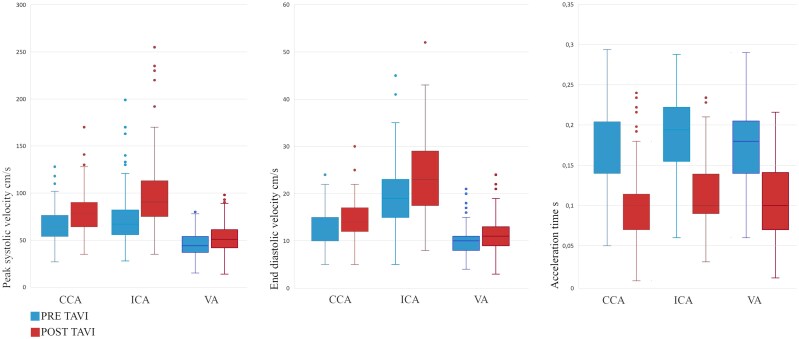
Effect of aortic stenosis on supra-aortic arteries duplex determined variables. Box plot of peak systolic velocity (left), end-diastolic velocity (middle), and acceleration time (right) detected on common carotid artery, internal carotid artery and vertebral artery prior and after transcatheter aortic valve implantation in a cohort of 105 patients diagnosed with severe aortic stenosis. CCA, common carotid artery; ICA, internal carotid artery; VA, vertebral artery; TAVI, transcatheter aortic valve implantation.

**Figure 2:**
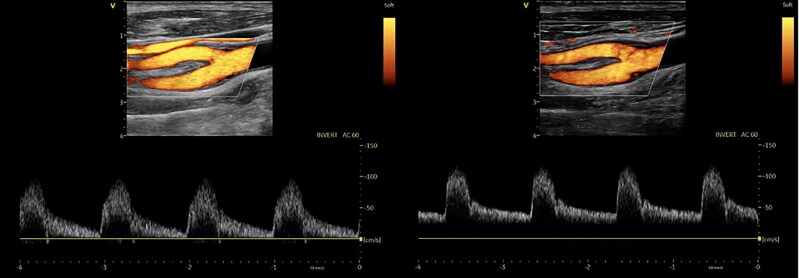
Carotid duplex assessment before and after transcatheter aortic valve implantation (TAVI). Representative colour Doppler with spectral curve of internal carotid artery of a patient diagnosed with severe aortic stenosis showing delayed upstroke (prolonged acceleration time), rounded curve of the waveform and a reduced magnitude before TAVI in (*A*) and after the procedure in (*B*) with normalization of the spectral curve.

Clinical and demographic characteristics are reported in *Table 1*. Among screened patients, 105 (42% men) with a median age of 80 years (IQR 76–85) met the inclusion criteria. The median interval time between pre- and post-TAVI supra-aortic blood flow indexes assessment was 5 days (IQR 3–9). Median post-operative length of stay was 4 days (IQR 2–7). None of the patients included in this study exhibited clinical signs of stroke within 30 days. Hemodynamic index changes and distribution details (IQRs) of examined supra-aortic vessels before and after TAVI are provided in *Table 2*. Following TAVI, there was a significant rise in median PSV across all examined sites: common carotid artery (CCA) from 64.5 to 78.0 cm/s (+24%; *P* < 0.01), ICA from 67.0 to 90.5 cm/s (+36%; *P* < 0.01), and VA from 44.0 to 51.0 cm/s (+17%; *P* < 0.01). Median EDV also increased: CCA from 12.0 to 14.0 cm/s (+12%; *P* < 0.01), ICA from 19.0 to 23.0 cm/s (+20%; *P* < 0.01), and VA from 10.0 to 11.0 cm/s (+18%; *P* < 0.01). In parallel, median AT decreased markedly at each site: CCA from 0.180 to 0.100 s (−44%; *P* < 0.01), ICA from 0.195 to 0.100 s (−41%; *P* < 0.01), and VA from 0.180 to 0.100 s (−36%; *P* < 0.01). Post-TAVI, stroke volume index and supra-aortic PSV and EDV exhibited concordant increases, whereas AT was reduced. A whisker plot highlighting the main results of the study is provided in *Figure 1*. Spectral Doppler examinations of the supra-aortic vessels after the procedures revealed no more AS related characteristics as delayed upstroke, rounded curve of the waveform and reduced magnitude in all cases as shown in *Figure 2*. Notably, according to these post-TAVI hemodynamic changes, four patients of this population, who pre-operatively were diagnosed with non-severe ICA stenosis based on velocity estimation, exceeded the threshold for severity after TAVI. A subgroup analysis was performed to assess whether the observed changes were comparable between the right and left sides of the supra-aortic trunks. This analysis showed that within-side pre–post changes were significant for all comparisons, and right–left comparisons of percentage change revealed no significant asymmetry for PSV and EDV in any vessel. For AT, a modest side difference emerged in the common carotid, with a greater reduction on the left (*P* = 0.01), whereas no side differences were observed in the internal carotid or vertebral arteries (*Table 3*).

**Table 1:** Demographics, cardiovascular risk factors, comorbidities, and aortic valve parameters of 105 patients diagnosed with severe AS who fulfilled the inclusion criteria

Variable	Value
Age, y, median (IQR)	80 (76–85)
Male, *n* (%)	44 (42)
Hypertension, *n* (%)	32 (30)
Dyslipidaemia, *n* (%)	34 (32)
DM, *n* (%)	9 (9)
CKD, *n* (%)	8 (8)
COPD, *n* (%)	8 (8)
CAD, *n* (%)	39 (37)
Previous AMI, *n* (%)	10 (10)
Previous CABG, *n* (%)	0 (0)
Previous PCI, *n* (%)	38 (36)
Aortic valve area, cm^2^, median (IQR)	0.7 (0.7–0.8)
Pre-operative median TG, mmHg (IQR)	44 (40–53)
Post-operative median TG, mmHg (IQR)	12 (8–14)
Pre-operative LVEF, % median (IQR)	56 (53–61)
Post-operative LVEF, % median (IQR)	59 (54–64)
Pre-operative SVi, mL/m² median (IQR)	45.1 (38.6–46.3)
Post-operative SVi, mL/m² median (IQR)	46.3 (39.4–48.7)
Mild paravalvular leak after TAVI, *n* (%)	40 (38)
Clinical signs of stroke at 30 days, *n* (%)	0 (0)

Data are presented as *n* (%) or median (inter-quartile range, IQR).

DM, diabetes mellitus; CKD, chronic kidney disease, defined by glomerular filtration rate < 60 mL/min; COPD, chronic obstructive pulmonary disease; CAD, coronary artery disease; AMI, acute myocardial infarction; CABG, coronary artery bypass grafting; PCI, percutaneous coronary intervention; TG, transaortic gradient; LVEF, left ventricle ejection fraction; SVi, stroke volume index; TAVI, transcatheter aortic valve implantation.

**Table 2:** Supra-aortic blood flow velocity indexes and their within-subject changes in the 105 patients included in the study

	Pre-TAVI, median [IQR]	Post-TAVI, median [IQR]	Δ (post−pre), median [IQR]	% change median	*P* (Wilcoxon)
CCA PSV (cm/s)	64.5 [54.0–76.0]	78.0 [64.5–90.0]	14.0 [4.0–21.0]	24.0%	<0.01
ICA PSV (cm/s)	67.0 [56.0–81.5]	90.5 [75.5–113.0]	22.5 [12.0–34.0]	35.8%	<0.01
VA PSV (cm/s)	44.0 [38.0–54.0]	51.0 [42.0–60.0]	7.0 [1.0–11.0]	17.5%	<0.01
CCA EDV (cm/s)	12.0 [10.0–15.0]	14.0 [12.0–17.0]	2.0 [0.0–3.0]	12.5%	<0.01
ICA EDV (cm/s)	19.0 [15.0–23.0]	23.0 [18.0–29.0]	4.0 [0.0–7.0]	20.0%	<0.01
VA EDV (cm/s)	10.0 [8.0–11.0]	11.0 [9.0–13.0]	2.0 [0.0–3.0]	18.2%	<0.01
CCA AT (s)	0.180 [0.140–0.204]	0.100 [0.070–0.114]	−0.065 [−0.118 to -0.044]	−44.1%	<0.01
ICA AT (s)	0.195 [0.155–0.222]	0.100 [0.090–0.138]	−0.072 [−0.120 to −0.037]	−41.2%	<0.01
VA AT (s)	0.180 [0.143–0.200]	0.100 [0.072–0.142]	−0.054 [−0.106 to −0.020]	−35.7%	<0.01

TAVI, transcatheter aortic valve implantation; IQR, inter-quartile range; CCA, common carotid artery; PSV, peak systolic velocity; ICA, internal carotid artery; VA, vertebral artery; EDV, end-diastolic velocity; AT, acceleration time.

**Table 3:** Comparison of within-subject percent changes between right and left sides

	Right: Δ% median	*P* (Right)	Left: Δ% median	*P* (Left)	*P* (Δ%R−Δ%L)
CCA PSV (cm/s)	+24.56	<0.01	+18.75	<0.01	0.09
ICA PSV (cm/s)	+35.29	<0.01	+36.36	<0.01	0.55
VA PSV (cm/s)	+16.17	<0.01	+18.42	<0.01	0.89
CCA EDV (cm/s)	+13.33	<0.01	+12.13	<0.01	0.17
ICA EDV (cm/s)	+19.38	<0.01	+20.00	<0.01	0.26
VA EDV (cm/s)	+18.18	<0.01	+18.18	<0.01	0.92
CCA AT (s)	−40.48	<0.01	−50.00	<0.01	0.01
ICA AT (s)	−40.00	<0.01	−43.59	<0.01	0.75
VA AT (s)	−36.00	<0.01	−34.00	<0.01	0.89

CCA, common carotid artery; PSV, peak systolic velocity; ICA, internal carotid artery; VA, vertebral artery; EDV, end-diastolic velocity; AT, acceleration time.

## Discussion

The study results demonstrate the abnormal flow pattern of supra-aortic trunks observed at the duplex assessment in the presence of a severe AS, decrease PSV and EDV in common, internal carotid and vertebral arteries and increase AT in the same arteries. These AS-related abnormalities were reversed after TAVI, a pattern consistent with prior observations by Cammalleri, who documented post-TAVI increases in carotid PSV and EDV together with a shortening of AT.^15^ So far, hemodynamic changes in the supra-aortic arteries with coexistence of AS have been evaluated with opposite results. In a retrospective registry,^16^ carotid artery waveform abnormalities were investigated reviewing acceleration time, peak velocity, and waveform contour of common, internal and external carotid arteries of 24 patients with various degrees of AS. The authors reported that acceleration time increase and peak velocity decrease correlated with the severity of the aortic valve disease. These data are consistent with our study supporting the concept that, in the presence of AS, the abnormal flow pattern results in a decreased blood flow velocity in the supra-aortic arteries. Whether this effect is reversible after relief of AS has been examined previously. In a retrospective comparison of PSV and EDV of the ICA of 92 patients prior and after surgical aortic valve replacement (SAVR) no difference could be observed.^4^ Of note, in that study, only 11% of patients underwent SAVR alone. Most patients underwent other cardiac procedures such as coronary artery bypass grafting, concurrent surgery on additional heart valves or other additional surgical procedures thus resulting in confounding bias. Furthermore, the mean interval between the two examinations was more than six months (182 ± 98 days). Conversely in a prospective study, evaluating 30 patients undergoing aortic valve replacement for AS, a post-procedural decreased AT and an increased PSV could be observed in all patients.^17^ In our study, we decided to isolate the effect of AS of supra-aortic arteries blood flow by limiting our assessment only to patients undergoing isolated TAVI. In these conditions, it is possible to avoid the consequences of open chest surgery and other cardiac procedures on the supra-aortic blood flow pattern. On this background, we decided to perform a post-operative duplex scan a few hours after TAVI because we hypothesized that supra-aortic arteries blood flow pattern is restored to normal briefly after TAVI. In accordance with this supposition, our study demonstrated an underestimation of ICA velocity indexes in presence of an AS. This effect could affect the diagnostic accuracy of duplex carotid artery pre-operative assessment.

### Study limitations and future directions

While the within-subject paired design mitigates between-patient confounding, the absence of a control cohort precludes robust causal attribution of the observed haemodynamic changes. The operator dependence of DUS is a further limitation; nevertheless, the highly selected cohort and standardized acquisition protocols likely attenuate measurement bias. Excluding patients with reduced left ventricular ejection fraction (LVEF < 50%) also constrains external validity, as this subgroup may present distinct hemodynamic patterns. Future multi-centre studies including broader and more heterogeneous populations, particularly those with impaired left ventricular function, are warranted to confirm and expand our findings, ultimately improving the generalizability and clinical applicability of our results.

### Clinical perspectives

The perioperative risk of stroke remains clinically relevant across coronary artery bypass grafting (CABG), SAVR, and TAVI.^18–20^ The presence of multiple coexisting causes makes studying the mechanism of stroke challenging.^21^ However, in TAVI, the dominant mechanism is embolic, driven by debris generated during device navigation through the aortic arch.^20,22^ In SAVR and CABG, most intra-operative strokes are thromboembolic and related to aortic manipulation or cannulation, whereas a minority (20–30%) are attributable to cerebral hypoperfusion under cardiopulmonary bypass with non-pulsatile flow and a mean arterial pressure of ∼40–60 mmHg.^18,19^ The latter mechanism may be exacerbated in the presence of bilateral severe carotid stenosis or a severe carotid stenosis with contralateral occlusion, scenarios in which, according to the European Society for Vascular Surgery, pre-operative carotid revascularization may be considered in selected patients before cardiac surgery.^23^ Since we observed that severe AS may lead to underestimation of asymptomatic ICA disease, further researches are needed to validate PSV thresholds that accurately detect ≥70% asymptomatic ICA stenosis in SAVR candidates, to identify patients with masked severe disease who might benefit from prophylactic carotid revascularization and potentially reduce perioperative stroke risk.

## Conclusions

Severe AS significantly decreases supra-aortic arteries blood flow and this effect can underestimate the grade of carotid artery stenosis. This study suggests that carotid ultrasound criteria to assess ICA stenosis severity should be re-evaluated in larger multi-centre studies to validate their predictive values in patients with concomitant AS and ICA stenosis.

The data underlying this article will be shared on reasonable request to the corresponding author.

## Supplementary Material

qyaf151_Supplementary_Data

## Lead author biography

Dr. Rocco Pasqua is a vascular surgeon trained at *Sapienza University of Rome*, where he also obtained his PhD in *Advanced Technologies in Surgery*. His clinical and research interests focus on cardiovascular disease and the application of emerging technologies in vascular and endovascular surgery. Dr. Pasqua’s work integrates a broad, multi-disciplinary perspective, encompassing related fields to promote a comprehensive and innovative approach to patient care and cardiovascular research.
