# Supplementary figures and images for: Hemodynamic changes in supra-aortic trunks after transcatheter aortic valve implantation at duplex ultrasound examination
Source: Eur Heart J Imaging Methods Pract. 2025 Dec 3;3(4):qyaf151. doi: 10.1093/ehjimp/qyaf151 (PMC12683244; doi:10.1093/ehjimp/qyaf151)

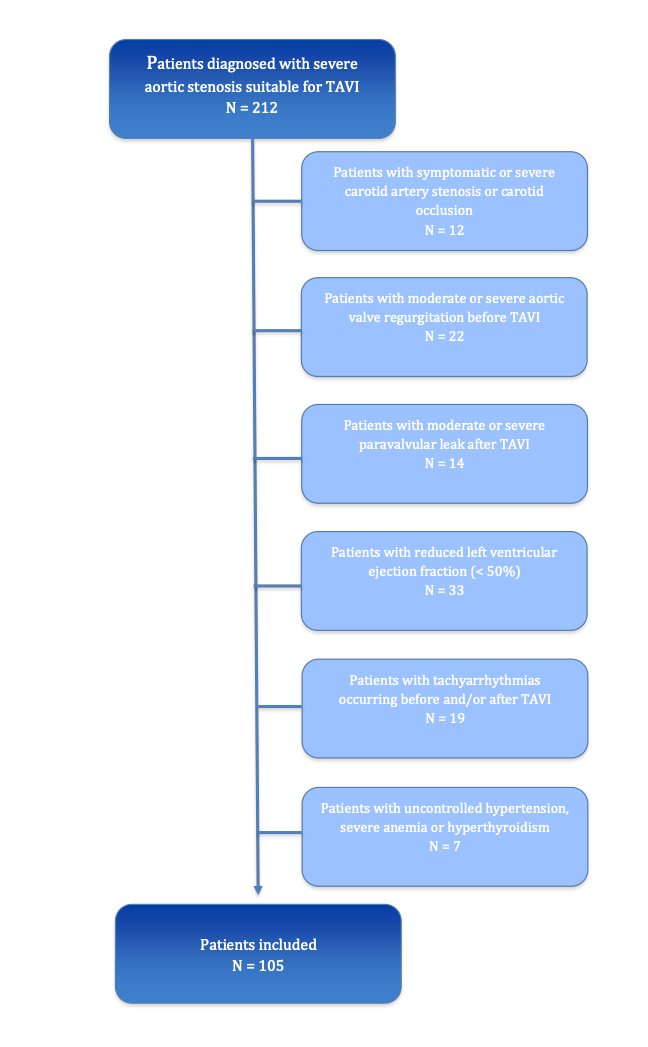

Supplement: qyaf151_Supplementary_Data [file qyaf151_supplementary_data.zip › Flow Chart.tif]
